# Prevalence of Malnutrition and Associated Factors Among the Elderly With Type 2 Diabetes Using MNA Form

**DOI:** 10.1155/jnme/2107146

**Published:** 2025-03-05

**Authors:** Thamina Rashid, Summaiyah Zia, Saba Mughal, Akhtar Ali Baloch, Mohammad Uzair Abdul Rauf, Syed Muhammad Hasan

**Affiliations:** ^1^National Institute of Diabetes and Endocrinology, Dow University of Health Sciences, Karachi, Pakistan; ^2^School of Public Health, Dow University of Health Sciences, Karachi, Pakistan

**Keywords:** elderly diabetics, Mini-Nutritional Assessment, nutritional status

## Abstract

**Objective:** This study has been conducted to identify the frequency of malnutrition and different factors associated with it among elderly people with Type 2 diabetes.

**Methods:** This cross-sectional study was conducted in the National Institute of Diabetes and Endocrinology (NIDE), DUHS, Karachi, between January 2023 and October 2023. A total of 325 elderly participants aged ≥ 60 years with Type 2 diabetes were included. Mini Nutritional Assessment (MNA) was used for data collection.

**Results:** Nutritional assessment of older diabetic patients according to the MNA revealed that 226 (69.5%) patients were at risk of malnutrition whereas 53 (16.3%) were malnourished. A total of 325 Type 2 diabetic patients were included in the study with a mean age of 65.7 ± 5.5 years, where 135 (41.5%) were male and 190 (58.5%) were female. Older patients (mean age: 65 years) were significantly more malnourished compared to those with normal nutrition (mean age: 62 years; *p*=0.021). Employed patients were less likely to have the risk of malnutrition as compared to housewives (*p*=0.005). Patients who had low family income were eight times more likely to be malnourished as compared to those who had better family income (*p*=0.003). It was also found that patients with low BMI and calf circumference will be more prone to be malnourished as compared to those with high levels of BMI (*p*=0.003) and calf circumference (*p*=0.013).

**Conclusion:** The majority of study participants were at risk of malnourishment, with associated factors such as rapid weight loss, poor health status, decline in physical activity, and food intake. Therefore, these findings highlight the importance of tailored interventions for at-risk individuals, including regular screening and nutritional support.

## 1. Introduction

Diabetes mellitus has become a worldwide growing problem. In 2019, approximately 19.3% of elderly patients were living with diabetes. It is expected that the number of people over 60 years of age with diabetes will reach 195.2 million by 2030 and 276.2 million by 2045. It is shown that managing Type 2 older people with diabetes can be challenging for healthcare professionals [1]. In Pakistan, elderly people over the age of 60 years are termed as senior citizens [2]. In Pakistan according to World Health Organization (WHO) findings in 1998, six percent of our population was over 60 years which are expected to double in 2025. In 2011, a study was conducted in Pakistan that showed that in 2023, the average life expectancy will be 72 years. In one of the leading hospitals in Karachi, 1 out of 5 inpatients are above 65 years. This situation puts a great challenge for resource-constraint society to give quality care to this vulnerable population [3].

The findings of the International Diabetes Federation in the year 2022 revealed that in Pakistan, 26.7% of adults are affected by diabetes, thus resulting in approximately 33,000,000 cases [4].

Aging significantly alters dietary patterns and nutrient intake, impacting elderly health [5, 6]. Due to malnourishment health suffers, hospital stays extend and mortality increases. Complications of diabetes and its associated diseases are most common in older people compared to their young population. Malnourishment has become a big reason for deteriorating nutritional status and difficulty in meeting nutritional needs [7].

These people have chronic diseases such as hypertension, dyslipidemia, and CVD due to which the nutritional requirement changes. Attainment of normal body weight in the elderly population may be found to be difficult compared to other age brackets [1]. Underweight elderly patients also face complications requiring care; overweight patients that cause insulin resistance also increase the risk of mortality and morbidity. Appetite changes, palatability, dietary limitations, depression, and feeling of loneliness all affect the dietary intake of people. Malnutrition causes undernutrition and micronutrient deficiencies. Protein-energy malnutrition is also a big concern and a contributing factor in weight loss. The functional status also decreases, which is called failure to thrive, which is indicated by 5% weight loss from initial weight, resulting from varied reasons including not taking the appropriate amount of calories and protein, low muscle mass, cognitive impairment, and dementia. Self-care activities also suffer due to the occurrence of social, domestic, and economic changes in the elderly population [8].

Although normal sugar control and health problems must be the first priority, individual preferences and quality of life must not be neglected. Nutrition therapy must be based on an individual approach, and evidence-based interventions are needed after screening for malnutrition to address nutrition-related problems [9].

In Pakistan, the healthcare system is not established, and a lack of resources and financial problems put extra pressure on elderly people and their caregivers. With diabetes and other comorbidities in elderly people, negligence in giving optimum nutrition support put an extra burden on the already fragile healthcare system and on family members due to deterioration in their health status [10].

In order to accurately diagnose and treat nutrition-related issues in older adults, comprehensive assessment and screening using standardized screening tools are required to identify individuals who are malnourished or at risk of malnutrition. This will help ensure that every elderly individual having diabetes receives appropriate standardized dietary counseling for their nutritional status. Moreover, limited studies have been conducted to assess malnutrition among elderly using MNA form in patients with Type 2 diabetes. Therefore, the objective of this study is to assess malnutrition status among them.

## 2. Methodology

### 2.1. Study Design and Population

This cross-sectional study was conducted at the National Institute of Diabetes and Endocrinology (NIDE), DUHS. Patients visiting the outpatient department (OPD) between January and October 2023 were included. A total of 325 patients with Type 2 diabetes were included in the study using convenience sampling. Only five patients who were unable to stand were excluded from the study.

The Raosoft online calculator was used to determine the sample size. The margin of error was kept at 5%, with a confidence interval of 95% and a response rate of 75%. The population size was taken based on a prior study which shows that 40.7% of the patients with diabetes in Pakistan are over 60 years or above [11].

Patients were informed about the research opportunity during their routine checkups. Voluntary participation was assured by all participants. A trained dietitian collected the data in Urdu language to ensure a better understanding. In addition to the questionnaire, demographic data such as age, gender, education, and family income were also taken.

### 2.2. Inclusion Criteria

• Patients diagnosed with Type 2 diabetes for at least 6 months in accordance with the WHO criteria.
• Patients aged 60 years or above were included.

### 2.3. Exclusion Criteria

• Patients with Type 1 diabetes or any other type of diabetes except Type 2 diabetes.
• Patients who were unable to stand up.
• Patients who did not give consent or who were unable to give consent.

### 2.4. Ethics, Consent, and Permissions

Data were collected from study participants after informed verbal consent, and full confidentiality was maintained throughout the study process. The patients were free to leave the question-and-answer session without giving any reason.

Institutional Review Board (IRB) of Dow University of Health Sciences approved the study (Ref: IRB-2928/DUHS/Approval/2023/163).

### 2.5. Questionnaire

A full Mini Nutritional Assessment (MNA) form, a validated questionnaire, was used for data collection [12]. The MNA form is a simple screening tool to help identify elderly patients who are malnourished or at risk of malnutrition. This tool has been used in several countries including Pakistan [13, 14], to assess malnutrition. Full MNA is an 18-item questionnaire that evaluates risk and identifies malnutrition in people most likely to benefit from early nutritional interventions. This tool is applicable to frail or healthy elderly. It consists of four simple sections: anthropometry, dietary assessments, clinical global assessment, and self-perception of nutritional status and overall health [15].

### 2.6. General Characteristics

The first section of the questionnaire consists of patient's general characteristics such as age, gender, ethnicity, occupation, family income, and duration of diabetes.

The second section consists of comorbidities and complications.

### 2.7. Anthropometric Measurements

For the assessment of BMI, weight (kg) and height (cm) were taken. The subject height was taken using a portable SECA 206 stadiometer while weight was analyzed using Omron HBF-514C scale. Measuring tape was used for mid-upper arm circumference (MUAC) and calf circumference (CC).

### 2.8. Statistical Analysis

Descriptive statistics was reported as frequency (percentage) for categorical variables and mean ± standard deviation and median (interquartile range) for continuous variables. All independent variables such as demographic, comorbidities, and anthropometric characteristics were compared with the nutritional status of patients using Kruskal–Wallis and chi-square/Fisher's exact test, where appropriate. Multinomial logistic regression analysis was carried out with normal nutrition status as a baseline and reported relative risk (RR) ratio to assess the association between independent factors and the nutritional status of patients. All *p* values < 0.05 were considered significant. SPSS Version 27 was considered for statistical analyses.

## 3. Results

A total of 325 Type 2 diabetic patients were included in the study. There were 135 (41.5%) male and 190 (58.5%) female with an average age of 65.7 ± 5.5 years which ranged between 60 and 90 years. The average MNA score was 20.1 ± 3.7. Nutritional assessment of older diabetic patients according to the MNA revealed that 226 (69.5%) patients were at risk of malnutrition whereas 53 (16.3%) were malnourished. Distribution of MNA screening and assessment items with the nutritional status of the patients are given in Table 1. Patients presented in the malnutrition group had higher median ages as compared to those in the normal nutritional group (65 vs. 62 years, *p* value = 0.021). A significantly greater number of patients who had a normal nutritional status were employed (26.1% vs. 9.4%, *p* value = 0.009) and had better family income (50% vs. 15.1%, *p* value < 0.001) than malnourished participants (Table 2). Those who were malnourished showed a higher tendency of having ketosis (47.2% vs. 19.6%, *p* value = 0.016), infection in the last 6 months (39.6% vs. 19.6%, *p*-value = 0.032), and foot injury (22.6% vs. 15.2%, *p* value = 0.043) as compared to well-nourished patients (Table 3).

It was found that most of the malnourished patients were underweight (15.1% vs. 0%) when compared with those who had normal nutritional status. Moreover, significantly higher number of patients who had normal nutritional status had higher BMI (*p* value < 0.001), mid-arm circumference (*p* value = 0.005), and CC(*p* value < 0.001) levels (Table 4). The association between nutritional status and several characteristics of diabetic patients was assessed and is shown in Table 5. Multinomial logistic regression analysis was run where the model was adjusted for those variables whose *p* values were less than 0.25. The multivariate regression model revealed that employed patients were less likely to have the risk of malnutrition as compared to housewives (*p* value = 0.005). Patients who had low family income were eight times more likely to be malnourished as compared to those who had better family income (*p* value = 0.003). It was also noted that patients with low BMI and CC were more prone to be malnourished as compared to those with high levels of BMI (*p* value = 0.003) and CC (*p* value = 0.013) (Table 5).

## 4. Discussion

Research studies have proven that both diabetes and old age are risk factors for malnutrition.

Due to poor dietary intake or malabsorption, diabetes-related malnutrition is common that results in deficiency of nutrients, causes multiple organ damage, and alters digestive, muscular, and immune functions that raise the prevalence of morbidity, mortality, and length of hospital stay [16].

In this study, we aimed at estimating the prevalence of malnutrition and its related factors among elderly diabetic patients visiting OPD of hospital in Karachi by using the MNA tool. MNA is a simple and validated tool for the assessment of malnutrition among elderly. This tool is recommended for routine nutritional assessment of elderly population in many countries [12, 17, 18]. To the best of our knowledge and after doing the literature search, there were no studies previously conducted in Pakistan to assess malnutrition in elderly with diabetes using MNA form.

In our study, 226 (69.5%) patients were found to be at risk of malnourishment while 53 (16%) were malnourished. A similar finding is also confirmed by a study conducted in Nigeria over 6-month period that reported the prevalence of malnutrition was high among diabetics when compared with nondiabetics [19]. However, a comparative review with low- to middle-income countries published in 2015 showed that 43.3% of Pakistani elderly are at risk of malnutrition [20]. Similarly, a study conducted in Pakistan at Agha Khan University Hospital, Karachi, reported 52% elderly had normal nutrition status [21].

Our findings demonstrated that as participants' age increases, the risk of malnutrition also increases. More than 67 years of elderly fall under the category of malnourishment and risk of malnourishment (*p* value 0.021). Other studies also favor this result that malnutrition is more prevalent among patients aged 70 or older [22].

Our study data showed that the risk of malnutrition was found to be predominant among elderly females than males, but the association was not found to be significant. Similar results were found in a study conducted in India [23]. However, malnourishment was found to be associated more in females than in males in some studies [24].

In our present study, employed patients were less likely to have the risk of malnutrition as compared to unemployed and housewives. In addition, patients who had low family income were eight times more likely to be malnourished as compared to those who had a better socioeconomic background (*p* value = 0.003). Similarly, a study conducted in India showed a significant association between poor socioeconomic status and malnutrition [25]. This highlights the importance of the relationship between socioeconomic status and nutritional outcomes.

Like other research studies, our results also showed that poor nutritional status is also associated with numerous illnesses including diabetes, hypertension [24, 26], coronary artery diseases, stroke, infection [16], foot injuries, dental problems, and eating disorders [24].

Many studies have linked dietary patterns that are poor in fruits and vegetables and rich in high glycemic index foods such as sweets and refined grains with increased risk of depression [27] and cardiovascular diseases [28, 29].

Our results indicated that among 226 elderly patients who were at risk of malnourishment, 78% were identified as obese and 21% were overweight. Likewise, out of 53 malnourished patients, 21% were obese while 14% were overweight.

Findings suggest that weight is not considered a reliable indicator to assess nutritional status among the elderly. Loss of muscle mass, poor appetite, inadequate food intake, and changes in body composition are also present in obese individuals. For accurate identification of malnutrition, dietary assessments, other anthropometric parameters, and biochemical markers are the reliable evidence [30, 31].

In addition, patients with low BMI were more prone to be malnourished as compared to those with high levels of BMI. Our results also show that no underweight individual was found among those with normal nutritional status. Diabetes can be a factor that also plays a role in the loss of muscle mass due to hyperglycemia [32].

Our present study showed that protein intake varied significantly among participants. Adequate protein consumption, from daily servings of dairy products, legumes and meat, fish, or chicken, was more common among patients with normal nutritional status. On the other hand, those who were malnourished or at risk had far lower protein intakes, thus highlighting the need of sufficient protein intake in preserving good nutritional health. Some studies have showed a positive association between malnutrition and inadequate protein intake [33, 34]. Based on the evidence, lower protein intake is linked with sarcopenia and frailty among the elderly that increases the prevalence of risk of mortality [35, 36].

Moreover, our study also highlighted several other factors which were prevalent among malnourished or at risk and was also evidenced by other studies such as reduced food intake and mobility [24], involuntary weight loss [7], psychological stress or acute disease [7], neurological problems [37, 38], pressure sores [39], polypharmacy [40, 41], less intake of fruits and vegetables [42], less consumption of fluids [43], and not satisfied with their health status [43].

Besides other factors, CC also showed a significant difference across nutritional status while MAC was not found to be significant. However, some studies have found a positive association between these measurements and nutritional status [24, 44, 45].

### 4.1. Limitations

As it is a single-centered study conducted in one city in Pakistan only, therefore the results may not reflect the data of the whole population. Another limitation includes the use of convenience sampling, which may have underrepresented groups such as higher income individuals, rural populations, and comorbidities potentially affecting the generalizability of the results.

### 4.2. Strength of the Study

This study's strengths include a large sample size of 325 individuals with Type II diabetes and a thorough evaluation of all the variables related to nutritional status. The validity of the results is increased by the use of a validated questionnaire to measure nutritional intake and health markers. Furthermore, identifying significant differences in protein consumption, psychological stress, and mobility restrictions advances our understanding of the complex nature of malnutrition in this demographic.

## 5. Conclusion

In conclusion, our study participants were found at an increased risk of malnutrition which is linked with rapid weight loss, poor health status, decline in physical activity, and food intake. Therefore, dietary education according to elderly patients' needs is required.

## Figures and Tables

**Table 1:** Mini Nutritional Assessment (MNA) items by nutritional status of Type 2 diabetic patients (*n* = 325).

Items	Normal nutrition	Risk of malnutrition	Malnutrition
	(*n* = 46)	(*n* = 226)	(*n* = 53)
Screening			
Declined food intake over past 3 months			
Severe	1 (2.2)	8 (3.5)	18 (34.0)
Moderate	2 (4.3)	101 (44.7)	24 (45.3)
No	43 (93.5)	117 (51.8)	11 (20.8)
Weight loss during last 3 months			
> 3 kg	1 (2.2)	13 (5.8)	27 (50.9)
Do not know	3 (6.5)	13 (5.8)	3 (5.7)
1–3 kg	12 (26.1)	98 (43.4)	21 (39.6)
No	30 (65.2)	102 (45.1)	2 (3.8)
Mobility			
Bed or chair bound	0	5 (2.2)	8 (15.1)
Able to get out of bed/chair but does not go out	2 (4.3)	25 (11.1)	16 (30.2)
Goes out	44 (95.7)	196 (86.7)	29 (54.7)
Psychological stress or acute disease in past 3 months			
Yes	5 (10.9)	167 (73.9)	47 (88.7)
No	41 (89.1)	59 (26.1)	6 (11.3)
Neurological problem (dementia/depression)			
Severe	1 (2.2)	4 (1.8)	7 (13.2)
Mild	8 (17.4)	158 (69.9)	40 (75.5)
No	37 (80.4)	64 (28.3)	6 (11.3)
Assessment			
Lives independently			
No	2 (4.3)	9 (4.0)	3 (5.7)
Yes	44 (95.7)	217 (96.0)	50 (94.3)
Takes > 3 prescription drugs/day			
Yes	12 (26.1)	114 (50.4)	33 (62.3)
No	34 (73.9)	112 (49.6)	20 (37.7)
Pressure sores/skin ulcer			
Yes	10 (21.7)	59 (26.1)	27 (50.9)
No	36 (78.3)	167 (73.9)	26 (49.1)
Full meals/day			
1	0	3 (1.3)	7 (13.2)
2	4 (8.7)	48 (21.2)	25 (47.2)
3	42 (91.3)	175 (77.4)	21 (39.6)
Protein intake			
≥ 1 serving of dairy products^ⴕⴕ^/day	9 (19.6)	108 (47.8)	30 (56.6)
≥ 2 servings of legumes or eggs/week	20 (43.5)	93 (41.2)	16 (30.2)
Meat, fish, or poultry/daily	17 (37.0)	25 (11.1)	7 (13.2)
Consume ≥ 2 servings of fruits and vegetables/day			
No	8 (17.4)	70 (31.0)	22 (41.5)
Yes	38 (82.6)	156 (69.0)	31 (58.5)
Fluid^¶^ consumption/day			
< 3 cups	0	4 (1.8)	4 (7.5)
3–5 cups	6 (13.0)	19 (8.4)	10 (18.9)
> 5 cups	40 (87.0)	203 (89.8)	39 (73.6)
Mode of feeding			
Unable to eat without assistance	0	6 (2.7)	5 (9.4)
Self-fed with some difficulty	3 (6.5)	5 (2.2)	3 (5.7)
Self-fed without any problem	43 (93.5)	215 (95.1)	45 (84.9)
Self-view of nutritional status			
Views self as being malnourished	3 (6.5)	161 (71.2)	44 (83.0)
Is uncertain of nutritional state	7 (15.2)	37 (16.4)	8 (15.1)
Views self as having no nutritional problem	36 (78.3)	28 (12.4)	1 (1.9)
Comparison of health status with others			
Not as good	10 (21.7)	166 (73.5)	44 (83.0)
Do not know	1 (2.2)	27 (11.9)	7 (13.2)
As good	18 (39.1)	20 (8.8)	1 (1.9)
Better	17 (37.0)	13 (5.8)	1 (1.9)

*Note: n* (%) are reported.

^ⴕⴕ^Milk, cheese, yogurt.

^¶^Water, juice, coffee, tea, milk.

**Table 2:** Sociodemographic characteristics with nutritional status of Type 2 diabetic patients (*n* = 325).

Characteristics	Normal nutritional	Risk of malnutrition	Malnutrition	*p* value⁣^∗^
	(*n* = 46)	(*n* = 226)	(*n* = 53)	
Age (years)	62 (60–67)	65 (60–70)	65 (62–71)	0.021
Gender				
Male	23 (50.0)	91 (40.3)	21 (39.6)	0.455
Female	23 (50.0)	135 (59.7)	32 (60.4)	
Education				
Uneducated	13 (28.3)	84 (37.2)	18 (34.0)	0.297
Primary	3 (6.5)	31 (13.7)	9 (17.0)	
Secondary	13 (28.3)	52 (23.0)	16 (30.2)	
Higher	17 (37.0)	59 (26.1)	10 (18.9)	
Ethnicity				
Urdu speaking	25 (54.3)	104 (46.0)	27 (50.9)	0.382
Sindhi	10 (21.7)	56 (24.8)	12 (22.6)	
Punjabi	4 (8.7)	16 (7.1)	8 (15.1)	
Pathan	5 (10.9)	42 (18.6)	4 (7.5)	
Balochi/other	2 (4.3)	8 (3.5)	2 (3.8)	
Employment				
Employed	12 (26.1)	19 (8.4)	5 (9.4)	0.009
Unemployed	10 (21.7)	83 (36.7)	19 (35.8)	
Housewife	24 (52.2)	124 (54.9)	29 (54.7)	
Family income (PKR)				
10–30	6 (13.0)	61 (27.0)	15 (28.3)	< 0.001
31–50	17 (37.0)	124 (54.9)	30 (56.6)	
> 50	23 (50.0)	41 (18.1)	8 (15.1)	
Duration of diabetes (years)				
0–10	25 (54.3)	111 (49.1)	22 (41.5)	0.359
11–20	12 (26.1)	83 (36.7)	19 (35.8)	
> 20	9 (19.6)	32 (14.2)	12 (22.6)	

*Note:* Median (*Q*_1_–*Q*_3_) and *n* (%) are reported.

⁣^∗^*p* value calculated by Kruskal–Wallis test and chi-square/Fisher's exact test.

**Table 3:** Comorbidities and complications with nutritional status of Type 2 diabetic patients (*n* = 325).

Characteristics	Normal nutritional	Risk of malnutrition	Malnutrition	*p* value⁣^∗^
	(*n* = 46)	(*n* = 226)	(*n* = 53)	
Dyslipidemia				
No	25 (54.3)	90 (39.8)	18 (34.0)	0.095
Yes	21 (45.7)	136 (60.2)	35 (66.0)	
Hypertension				
No	21 (45.7)	66 (29.2)	19 (35.8)	0.083
Yes	25 (54.3)	160 (70.8)	34 (64.2)	
Coronary heart disease				
No	41 (89.1)	188 (83.2)	40 (75.5)	0.192
Yes	5 (10.9)	38 (16.8)	13 (24.5)	
Heart failure				
No	45 (97.8)	219 (96.6)	50 (94.3)	0.621
Yes	1 (2.2)	7 (3.1)	3 (5.7)	
Stroke				
No	45 (97.8)	218 (96.5)	49 (92.5)	0.406
Yes	1 (2.2)	8 (3.5)	4 (7.5)	
Numbness in hands and feet				
No	26 (56.5)	78 (34.5)	25 (47.2)	0.010
Yes	20 (43.5)	148 (65.5)	28 (52.8)	
Hypoglycemia in past 6 months				
No	35 (76.1)	148 (65.5)	31 (58.5)	0.183
Yes	11 (23.9)	78 (34.5)	22 (41.5)	
Ketosis				
No	37 (80.4)	145 (64.2)	28 (52.8)	0.016
Yes	9 (19.6)	81 (35.8)	25 (47.2)	
Infection in last 6 months				
No	37 (80.4)	173 (76.5)	32 (60.4)	0.032
Yes	9 (19.6)	53 (23.5)	21 (39.6)	
Current foot injury				
No	39 (84.8)	203 (89.8)	41 (77.4)	0.043
Yes	7 (15.2)	23 (10.2)	12 (22.6)	

*Note: n* (%) are reported.

⁣^∗^*p* value calculated by chi-square/Fisher's exact test.

**Table 4:** Anthropometric characteristics with nutritional status of Type 2 diabetic patients (*n* = 325).

Characteristics	Normal nutritional	Risk of malnutrition	Malnutrition	*p* value⁣^∗^
	(*n* = 46)	(*n* = 226)	(*n* = 53)	
BMI (kg/m^2^)				
Underweight	0	5 (2.2)	8 (15.1)	< 0.001
Normal	1 (2.2)	15 (6.6)	10 (18.9)	
Overweight	7 (15.2)	29 (12.8)	14 (26.4)	
Obese	38 (82.6)	177 (78.3)	21 (39.6)	
Mid-arm circumference (cm)				
< 21	0	12 (5.3)	8 (15.1)	0.005
21–22	1 (2.2)	14 (6.2)	6 (11.3)	
> 22	45 (97.8)	200 (88.5)	39 (73.6)	
Calf circumference (cm)				
< 31	9 (19.6)	78 (34.5)	31 (58.5)	< 0.001
≥ 31	37 (80.4)	148 (65.5)	22 (41.5)	

*Note: n* (%) are reported.

⁣^∗^*p* value calculated by chi-square/Fisher's exact test.

**Table 5:** Relative risk ratio of malnutrition (compared to normal nutritional) for Type 2 diabetic patients (*n* = 325).

Characteristics	Univariate analysis				Multivariate analysis			
	Risk of malnutrition		Malnutrition		Risk of malnutrition		Malnutrition	
	RR ratio (95% CI)	*p* value	RR ratio (95% CI)	*p* value	RR ratio (95% CI)	*p* value	RR ratio (95% CI)	*p* value
Age (years)	1.07 (0.99, 1.14)	0.056	1.10 (1.02, 1.19)	0.013	1.07 (1.00, 1.16)	0.050	1.08 (0.98, 1.18)	0.088
Gender								
Male	0.67 (0.35, 1.27)	0.224	0.65 (0.29, 1.45)	0.301	—	—	—	—
Female	Ref		Ref					
Education								
Uneducated	1.86 (0.84, 4.12)	0.126	2.35 (0.81, 6.77)	0.113	—	—	—	—
Up to secondary	1.49 (0.69, 3.19)	0.300	2.65 (0.97, 7.23)	0.056	—	—	—	—
Higher	Ref		Ref					
Employment								
Employed	0.30 (0.13, 0.71)	0.006	0.34 (0.10, 1.12)	0.076	0.25 (0.10, 0.66)	0.005	0.26 (0.06, 1.01)	0.051
Unemployed	1.60 (0.73, 3.53)	0.239	1.57 (0.61, 4.01)	0.344	1.18 (0.50, 2.75)	0.702	0.81 (0.28, 2.34)	0.703
Housewife	Ref		Ref		Ref		Ref	
Family income (PKR)								
10–30	5.70 (2.13, 15.22)	0.001	7.18 (2.07, 24.89)	0.002	6.42 (2.24, 18.35)	0.001	8.41 (2.08, 33.94)	0.003
31–50	4.09 (1.99, 8.40)	< 0.001	5.07 (1.86, 13.80)	0.001	5.02 (2.28, 11.06)	< 0.001	7.49 (2.39, 23.41)	0.001
> 50	Ref		Ref		Ref		Ref	
Duration of diabetes (years)								
0–10	1.24 (0.53, 2.94)	0.612	0.66 (0.23, 1.86)	0.432	—	—	—	—
11–20	1.94 (0.74, 5.05)	0.172	1.18 (0.38, 3.66)	0.765	—	—	—	—
> 20	Ref		Ref					
BMI (kg/m^2^)								
Underweight/normal	4.29 (0.55, 32.97)	0.161	32.57 (4.05, 261.50)	0.001	4.36 (0.48, 39.20)	0.188	30.39 (3.13, 295.16)	0.003
Overweight	0.88 (0.36, 2.18)	0.798	3.61 (1.26, 10.36)	0.017	0.87 (0.32, 2.32)	0.784	3.61 (1.13, 11.49)	0.029
Obese	Ref		Ref		Ref		Ref	
Mid arm circumference (cm)								
≤ 22	5.85 (0.77, 44.24)	0.087	16.15 (2.03, 128.47)	0.009	2.74 (0.32, 23.08)	0.352	3.33 (0.35, 31.36)	0.292
> 22	Ref		Ref		Ref		Ref	
Calf circumference (cm)								
< 31	2.16 (0.99, 4.71)	0.052	5.79 (2.33, 14.40)	< 0.001	1.92 (0.79, 4.63)	0.146	3.90 (1.33, 11.40)	0.013
≥ 31	Ref		Ref		Ref		Ref	

*Note:* Multivariate model was adjusted for those variables whose *p* value < 0.025 in univariate analysis.

Abbreviations: CI = confidence interval, RR = relative risk.

## Data Availability

The data that support the findings of this study are available from the corresponding author upon reasonable request.
